# Delay/Disruption Tolerant Network-Based Message Forwarding for a River Pollution Monitoring Wireless Sensor Network Application

**DOI:** 10.3390/s16040436

**Published:** 2016-03-25

**Authors:** Carlos Velásquez-Villada, Yezid Donoso

**Affiliations:** Systems and Computing Engineering Department, Universidad de los Andes, 111711 Bogotá D.C., Colombia; ydonoso@uniandes.edu.co

**Keywords:** disruption-tolerant, delay-tolerant, WSN, delivery probability, opportunistic forwarding, pollution monitoring, rural telecommunications

## Abstract

Communications from remote areas that may be of interest is still a problem. Many innovative projects applied to remote sites face communications difficulties. The GOLDFISH project was an EU-funded project for river pollution monitoring in developing countries. It had several sensor clusters, with floating WiFi antennas, deployed along a downstream river’s course. Sensor clusters sent messages to a Gateway installed on the riverbank. This gateway sent the messages, through a backhaul technology, to an Internet server where data was aggregated over a map. The communication challenge in this scenario was produced by the antennas’ movement and network backhaul availability. Since the antennas were floating on the river, communications could be disrupted at any time. Also, 2G/3G availability near the river was not constant. For non-real-time applications, we propose a Delay/Disruption Tolerant Network (DTN)-based solution where all nodes have persistent storage capabilities and DTN protocols to be able to wait minutes or hours to transmit. A mechanical backhaul will periodically visit the river bank where the gateway is installed and it will automatically collect sensor data to be carried to an Internet-covered spot. The proposed forwarding protocol delivers around 98% of the messages for this scenario, performing better than other well-known DTN routing protocols.

## 1. Introduction

The “GOLDFISH—Detection of Watercourse Contamination in Developing Countries using Sensor Networks” project was an Information and Communication Technologies (ICT) project funded from 2012 to 2015 by the European Union (EU). It was developed by a consortium of 11 universities and organizations from Poland, France, Sweden, Croatia, Slovakia, Colombia, and Bolivia. The GOLDFISH project worked with the Raspberry Pi platform to integrate all its components and developed the sensors technology, the communication modules, the Central Management and Monitoring platform, and the communication algorithms. In the project’s extension, an energy generation technology and a pollutant locator application were added [1].

**Figure 1:**
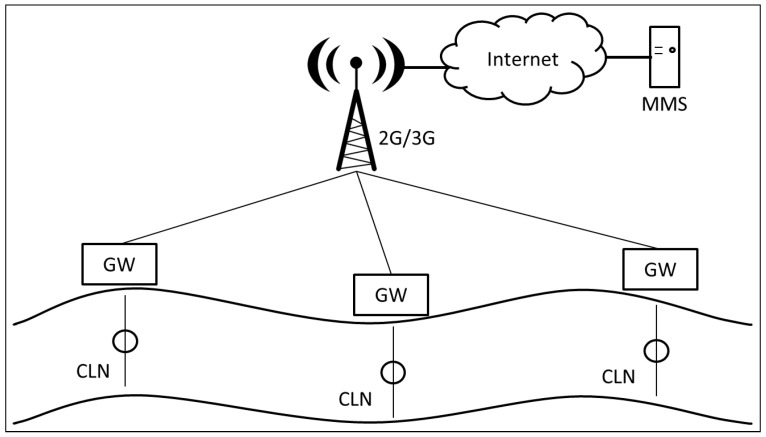
GOLDFISH project description. Sensor Nodes (CLN), Management and Monitoring Station (MMS), and Gateways (GW) are depicted.

**Figure 2:**
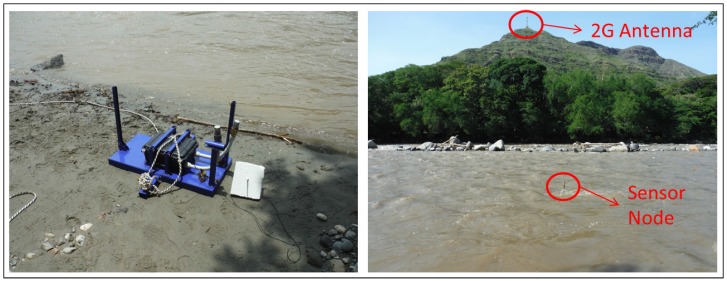
GOLDFISH project running in Colombia, 2015.

The GOLDFISH project was designed, as can be seen in Figure 1, to sense river pollution with some embedded sensors, and to send these data to a Gateway (GW) on the river’s shore. The GW sent sensor data to the central Management and Monitoring Station (MMS) via VPN through the Mobile network. The communication between the nodes in the river and between the nodes and the GW was achieved via WiFi (IEEE 802.11b) with a floating antenna incorporated in the sensor node design (see Figure 2). The GW had 2G/3G communication capabilities and it sent the sensor data through this channel. Once the MMS had the sensor data, it could show anomalies or alarms for higher than expected levels of pollutants. The system was tested in Europe and in Colombia. However, for tests in Colombia, the system had communication failures. The communication from the GW to the MMS did not work. There was a cellular antenna near the test site (it can be seen in Figure 2), but the data throughput varied between 20 and 95 kbps, and the data delay varied from 450 ms to 2.12 s to reach a server in Bogotá D.C. (100 km from the test site). In addition, the VPN to the MMS had to be created with a server in Warsaw. After two days of testing, this communication part of the project (GW to MMS) did not work. These problems were probably due to the lack of availability of 3G channels at the test site and the high delays experienced in the 2G channel available. This might have caused the VPN request to expire before connecting with the MMS or before the data could be sent to the MMS.

**Figure 3:**
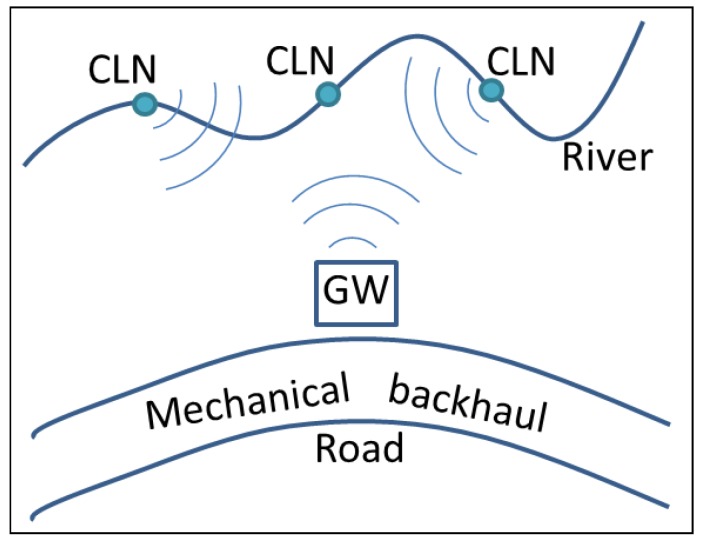
Modified GOLDFISH project. Cluster Nodes (CLN) and Gateway (GW) are depicted.

To solve the GOLDFISH project communication problem and to deal with even more restricted communication capabilities that can be present on more remote sites, we are proposing a Delay/Disruption Tolerant Network (DTN) that can use local transportation facilities to send and receive messages to Internet servers for non real-time and non-time-critical applications. The main idea is to send the sensor node information to the GW when possible or to a neighboring sensor node if it has a better probability to deliver the information to the GW. Sensor nodes (CLN) and the GW will store all data until they can reach the next hop en route to their destination. For the GW, it will be a mobile node that will transport these data to an Internet connected site, where they will be sent to the MMS (See Figure 3). Sensor nodes can communicate with each other and with the GW and learn who are their neighbors, how often they meet, and calculate the delivery probabilities through other nodes, so they can make decisions about which node will be the next hop in their transmission. The GW will receive all messages from the sensor nodes and will wait until a mobile node (MAP) comes by and collects the data. These MAPs will be mechanical vehicles that will, eventually or periodically, visit the site and contact the GW. When the GW encounters a MAP it will calculate the MAP’s delivery probability, and will decide if it gives the MAP all the data or if it should wait for the visit of another MAP with a better delivery probability. The GW can calculate these delivery probabilities based on historical information of contacts and the delivery probabilities given by these same MAPs. More details about the solution will be given in Section 3.

Section 2 will present some previous and related work for this kind of network. The proposal mentioned above, as well as its detailed algorithm, are presented in Section 3. Simulation results will be shown and analyzed in Section 4. Finally, conclusions will be presented in Section 5.

## 2. Previous and Related Works

In this project we are looking for a connectivity solution in a remote, usually disconnected site, near a river in underdeveloped countries, to connect a Wireless Sensor Network (WSN) solution for pollution monitoring. A possible solution for this scenario are Delay/Disruption Tolerant Networks (DTN). DTNs have been used in rural applications for several years. Although DTNs started as an alternative to spatial communications led by NASA, they have expanded to several applications in Earth-based communications; for example, Wireless Sensor Networks, Remote and Extreme Environments Monitoring, and Rural Communications. The following subsections describe the DTN Architecture, the Bundle Protocol, some routing and forwarding algorithms, and some rural connectivity technologies.

### 2.1. DTN Architecture and Bundle Protocol

The DTN Architecture [2] of 2007 describes an architecture for networks with special conditions where traditional protocols (TCP/IP) will not work. This architecture defines a message-oriented layer over the traditional transport layer, the Bundle Layer. The Bundle Layer establishes a store-and-forward kind of network with persistent storage as a measure against network disconnections, and uses Uniform Resource Identifiers (URI) [3] as a naming scheme. Applications can deliver and read Application Data Units (ADUs) to and from the Bundle Layer, which will encapsulate them into Bundles. These Bundles will be stored in persistent storage, and the responsibility of delivery can be transferred with the Bundle, creating a kind of distributed end-to-end reliability. The DTN Architecture allows unicast, anycast, and multicast communications, and it can also use priorities. Finally, the DTN Architecture can take advantage of continuous, scheduled, predicted, and opportunistic connectivity.

The Bundle Protocol [4] defines the exchange of messages (Bundles) between DTN nodes. The Bundle Protocol works as an application in the traditional networking model, since it seats on top of the transport layer, communicating DTN applications between DTN nodes. Its inner working looks like a communication between a couple of neighboring nodes for normal protocols, but this communication can include the transfer of custody and responsibility of delivery of a message. DTN nodes, then, can store Bundles until they have the opportunity to send them to their destination or to another node that can deliver them. In normally disconnected or high-delay networks, this storage can lasts for minutes, hours or days. The Bundle Protocol is independent of networking protocols in the layers below its implementation, and applications should be DTN-compliant or at least know what to do when a message arrives long after its request.

### 2.2. Rural Connectivity Technologies

Recently, there have been many initiatives to connect the unconnected half of the world. These unconnected citizens are, primarily, located in rural areas in developing countries. Main players in the IT world, like Google, Facebook and Intel, are heading these initiatives. In the next paragraphs we will review a couple of their projects and a DTN-Based rural connectivity technology, the DakNet project.

Google’s Loon project [5] is a Google initiative to bring Internet to underdeveloped and remote regions using stratospheric, solar-powered balloons. It uses base stations on Earth, below the balloon’s trajectory, and it creates a mesh network between the balloons to transfer messages to these ground base stations with Internet connectivity to transmit replies and requests. Users can connect to the balloons via LTE (Long Term Evolution) devices through a local carrier’s infrastructure.

Facebook’s Internet.org [6] uses local infrastructure, along with satellite communications and unmanned autonomous vehicles (UAV), to deliver a limited version of Internet to rural areas not currently covered or where access to Internet is very expensive. Users need an Internet.org account or to be affiliated with Facebook’s local mobile partner and they will have access to Facebook, local news, employment, health, and weather websites.

The main example of DTN use for Rural Connectivity is DakNet [7,8]. DakNet is a technology based on DTNs that uses buses and local transportation infrastructure to transport messages between small and remote towns and Internet. DakNet has been in use for several years in India and Bangladesh and it uses PROPHET [9] (see next subsection for more details on this protocol) as its routing protocol. DakNet establishes small kiosks in rural towns with no Internet connectivity that allow people to send emails and request information offline, and then, using the transportation infrastructure that comes into town, it transports these requests to Internet using the connection at the bus station in the nearest city and delivers their answers when the transport returns to town.

There are alternatives to use DTNs in city environments, like the one proposed in [10]. In this proposal, DTNs can be used to collect sensor data for Smart Cities in an Urban Automation Network (UAN). The nodes can be installed in different parts of the city, and a Garbage truck or a bus will eventually (at least once a day, according to the authors) collect the sensor information. In the paper, DTNs are compared with different alternatives and conclude that they are an efficient solution for sporadic transmissions.

### 2.3. DTN Routing and Forwarding Algorithms

Epidemic routing [11] is one of the earliest opportunistic routing protocols. In its basic form, a node running epidemic routing replicates the messages it needs to send to every neighbor it meets. Then, each message is replicated by those neighbors to their neighbors, until, eventually, the message reaches its destination. Epidemic routing can guarantee the delivery of all messages if enough resources are available. However, it can have problems with buffer and bandwidth management, as the messages can quickly exhaust the available capacity.

Spray and Wait routing [12] is an evolution of epidemic routing. A node running Spray and Wait also replicates the messages it needs to send, but it does so in two phases. The first phase, Spray, replicates a limited number of copies using one of two schemes (Source or Binary). The second phase, Wait, just transmits the message when it encounters its destination, stopping the replication process.

BUBBLE routing [13] is a protocol that exploits social features of the nodes communicating in a intermittently connected network. It uses labels to know to which communities or subcommunities the nodes belong to, and it also uses the centrality of the nodes to determine a ranking and take forwarding decisions.

The most known routing protocol for DTNs is PROPHET [9]. PROPHET stands for a Probabilistic Routing Protocol using History of Encounters and Transitivity. A node running PROPHET uses a history of contacts to assign delivery probabilities to nodes it meets and also to nodes its contacts can reach (transitivity). These probabilities decay over time so nodes can predict delivery probabilities to remote nodes.

Authors in [14] propose MPAD (Mobility Prediction-based Adaptive Data gathering protocol), a routing protocol that replicates messages and sends them to high delivery probability nodes. This protocol manages nodes’ queues based on the messages’ time to live, and it manages forwarding decisions based on direction movement regarding sink position. Authors assume that nodes have GPS or positioning capabilities and that nodes can determine where the sink is.

Other social-based routing protocols for DTNs are SimBet [15] and PeopleRank [16]. These protocols use some measurements or estimations about nodes’ centrality, betweenness, similarity, or social distance to rank or qualify neighboring nodes and decide whether or not to pass them the message.

Finally, other hybrid routing protocols are RAPID [17], SMART [18], and LSF [19]. RAPID (Resource Allocation Protocol for Intentional DTN) qualifies neighboring nodes with a utility function and replicates its message to the best qualified nodes. SMART (Selectively MAking pRogress Towards delivery) creates communities of nodes called companions and replicates the messages to the destination companions and, after a time threshold, the nodes will behave like binary Spray-and-Wait. LSF (Last Seen First spraying) passes several copies of a message (sprays) to a neighboring node with a more recent contact time to the destination.

Table 1 summarizes the classification of these previously described protocols. More routing protocols and other classifications can be found in reviews [20,21,22]. Our algorithm uses ideas from some of the protocols described in this section. It can be considered a hybrid protocol, since it has social, probabilistic, and replication characteristics. In the next section we will give more details about it.

## 3. A Non-Real-Time DTN-Based Forwarding Algorithm

### 3.1. A New Multi-Objective Mathematical Model

In this section we introduce a new mathematical model, built over our previous research [23], that will describe the message forwarding from sensor nodes, through Gateways (GWs) and Mobile Access Points (data mules or MAPs), to the Internet Connected Server (ICS) where the sensor data will be sent to the Central Management and Monitoring Platform (MMS). This mathematical model assumes that the communication will only take place from sensor nodes (source of information) to the MMS (the ICS will be the destination).

**Figure 4:**
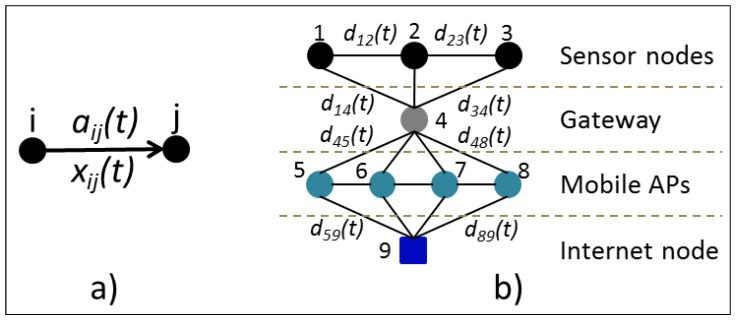
Problem graph. (**a**) Availability probability and data flow from node *i* to node *j*; (**b**) Network graph with delivery probabilities varying through time. AP: Access Point.

The mathematical model is presented in Equations (2)–(8). We are proposing a model for a network where their nodes can change their availability at any given time. The availability changes for the sensor nodes come from the fact that the GOLDFISH project uses microwave communication (IEEE 802.11b) and antennas floating over water. The antennas movement by the river flow can cause the water to cover them or tilt them to an angle where communication with the GW is no longer possible. Also, communication from the GW to the MMS is not always possible through the mobile cell network, so we will instead use local transportation means like buses or boats to recover sensor data from the GW and take them to the nearest ICS where it could be transferred to the MMS. The availability of the MAPs will change depending on their physical closeness with the GW. We assume that these vehicles transporting the MAPs will periodically or eventually visit the site where the GW is located. Figure 4 shows a graphical representation of the problem, where the availability probability $a_{ij}\left(t\right)$ between each pair of nodes depends on the existence (at any given time) of a physical link between them, and the delivery probability $d_{ij}\left(t\right)$ depends on the availability probability and the knowledge of the next node about a path to the destination, see Equation (1). In Equation (1), node *k* is the destination node, node *i* is the source node, and node *j* is an intermediary node. If the node *j* can deliver the message to node *k* at time *t*, the delivery probability $d_{jk}\left(t\right)$ will be equal to $a_{jk}\left(t\right)$.

(1) $$\begin{array}{c} d_{ij}\left(t\right)=a_{ij}\left(t\right)d_{jk}\left(t\right) \end{array}$$

**Figure 5:**
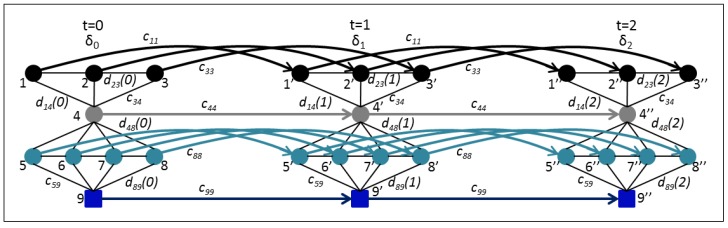
Problem graph through time. Temporal links go from left to right and represent nodes’ buffers.

Our mathematical model will have a set of nodes (*N*) where some nodes will be information-originating ones (sensor nodes in the river), others will be transit nodes (GW and MAPs), and one will be the destination node (the ICS in contact with the MMS). Each of these nodes will have a demand or an offer modeled by the parameter $b_{i}\left(t\right)$ (an integer vector) that can change over time. There is also a set of paths (*P*) that represent possible paths over the network through time. Paths are given by possible physical connections, so a path can be created through contacts of several nodes even if they happen at different times. The delivery probabilities $d_{ij}\left(t\right)$ are each of the probabilities that a node *i* can reach its destination through a node *j*. They depend on the link availability probability and the next node delivery probability to the destination. Equation (1) describes the delivery probability for a node *i* to reach a node *k* through a node *j*. Links between nodes have a given capacity modeled by $c_{ij}$, and nodes also have buffers to store data for some time, modeled by $c_{ii}$. We are assuming these capacities to be constant over time. The decision to send data over a link or store it until later is modeled through the positive integer variable $x_{ij}\left(t\right)$. This decision variable models the amount of data that is sent through a link at any given time and it is used to maximize the amount of data flowing through the highest delivery probability paths and minimize the time used to get the data to their destination. Table 2 shows all parameters and sets used in the model. Figure 5 shows the network graph expanded through time.

The multiobjective mathematical model maximizes the data flow through the paths with the highest delivery probabilities over time and at the same time it tries to minimize the delivery time for these messages in the network (see Equations (2) and (3). Constraints are described in Equations (4)–(7). Equations (4) and (5) are flow and balance constraints that require demands to be met, so all messages are delivered to their destination through one or several time steps. Equation (4) states that at time zero (0) what a node sends to other neighboring nodes and stores in its buffer should be equal to the offer of the node ($b_{i}\left(t\right)$) plus the messages received from other nodes. Equation (5) is a generalization of Equation (4) for times greater than zero. It takes into account the messages that are stored in a nodes’ buffer from the previous time. Equations (6) and (7) are capacity constraints that require that for any given time, information flow over links and stored in buffers must be equal to or below these links and buffers’ capacities. Finally, constraint in Equation (8) states that the decision variable $x_{ij}\left(t\right)$ is a positive integer defined over all links for all paths.

Objective Functions

(2) $$\begin{array}{c} \max\prod_{(i,j)\in P}d_{ij}\left(t\right)x_{ij}\left(t\right) \end{array}$$

(3) $$\begin{array}{c} \min\sum_{(i,j)\in P}\sum_{t\in T}x_{ij}\left(t\right)\delta_{t} \end{array}$$

Constraints

(4) $$\begin{array}{c} \sum_{j\in N}x_{ij}\left(t\right)-\sum_{j\in N}x_{ji}\left(t\right)+x_{ii}\left(t\right)=b_{i}\left(t\right)\;\forall \left(i,j\right)\in E,i\neq j,t=0 \end{array}$$

(5) $$\begin{array}{c} \sum_{j\in N}x_{ij}\left(t\right)-\sum_{j\in N}x_{ji}\left(t\right)+x_{ii}\left(t\right)-x_{ii}\left(t-1\right)=b_{i}\left(t\right)\;\forall \left(i,j\right)\in E,i\neq j,t\geq 1 \end{array}$$

(6) $$\begin{array}{c} x_{ij}\left(t\right)+x_{ji}\left(t\right)\leq c_{ij}\delta_{t}\;\forall \left(i,j\right)\in E,i\neq j \end{array}$$

(7) $$\begin{array}{c} x_{ii}\left(t\right)\leq c_{ii}\;\forall i\in N \end{array}$$

(8) $$\begin{array}{c} x_{ij}\left(t\right)\in Z_{\geq 0}\;\forall \left(i,j\right)\in E,p\in P \end{array}$$

### 3.2. Distributed Forwarding Algorithm

Nodes have to make forwarding decisions with local and limited information. Thus, in our solution, nodes use a distributed forwarding algorithm based on historical and social information. Each node meets its neighbors and gives them an availability probability that will decay over time and will increase when they meet again. Also, when a node meets a neighbor, they share their delivery probabilities to the destination node. Sensor nodes will start with a delivery probability near zero, so that nodes will prefer to store their messages until they contact the Gateway or another node that had been in contact with the Gateway, updating its delivery probability. They will also update their delivery probabilities through time, diminishing them as time goes by without further contacts with the same node. This updating will follow Equation (9). MAPs and the Gateway will start with a delivery probability near 0.9, since they have the links that must be transited on the network. Sensor nodes can use other sensor nodes to deliver their messages to the Gateway if they have a better delivery probability and are available to receive the messages. The Gateway will also update its delivery probability according to its knowledge of the network, giving higher delivery probabilities to MAPs closer to a scheduled or predicted encounter and closer, in time, to deliver the message to the Internet Connected Node.

(9) $$\begin{array}{c} d_{ij}\left(t+v\right)=d_{ij}{\left(t\right)}^{v} \end{array}$$

**Figure 6:**
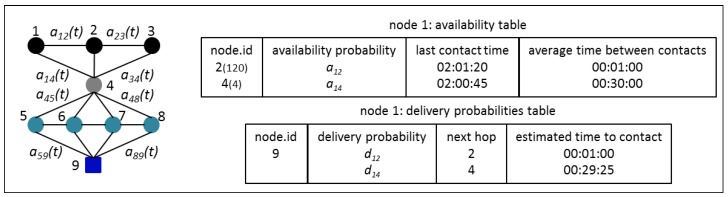
Availability table and delivery probabilities table for each node.

Nodes have to save the last contact time, the number of contacts, and calculate the mean time between contacts for every neighboring node. Based on this mean time between contacts, nodes will update the availability probabilities, giving them the maximum value when the contact should be close in time and diminishing them exponentially using Equation (9), where *v* is the number of time values since the last update. If the node has multiple neighbors with paths to the destination, then it can make the decision to wait for a certain node to become available or to deliver its message through an already available node, depending on their delivery probabilities and mean time between contacts. We are also using a replication option, where a source node can create up to one copy of a message (there will exist up to two identical messages in the network at any given time) and give a copy to each of the two neighboring nodes with the highest delivery probabilities. Sensor nodes, then, can use other nodes to reach the Gateway. The Gateway can use one of several MAPs that will pass nearby to deliver their messages. The messages will eventually reach their destination, the Central Management and Monitoring Platform (MMS). The Distributed Forwarding Algorithm is shown in pseudo-code in Algorithm 1. Figure 6 shows the tables that the nodes will create to keep their availability and delivery probabilities to their neighbors and messages destinations. The number between parentheses in the availability table is the number of contacts with each node.
**Algorithm 1** Distributed algorithm for every node in the network.1:node start (initialization)2:broadcast ID3:listen for neighbors’ IDs4:wait for messages()5:**if** message received **then**6:    save neighbors’ IDs and contact time in neighbors list7:    update mean time between contacts8:    update availability and delivery probabilities9:    update number of contacts with this neighbor10:**if** neighbor requests delivery probability **then**11:    **if** final destination = own ID **then**12:        send delivery probability 113:    **else** **if** destination known **then**14:        return delivery probability (not through requesting node)15:    **else** **if** destination unknown **then**16:        do not reply17:**else** **if** neighbor sends a message **then**18:    **if** it is for me **then**19:        read message20:    **else**21:        store message and send it to next node22:**else** **if** node has message to send **then**23:    ask for delivery probabilities to destination24:    send a copy of the message to the two best delivery probability neighbors25:    **if** no delivery probabilities **then**26:        store message in buffer until a path to destination appears

## 4. Results and Discussion

The results from the Distributed Forwarding Algorithm (DFA) were obtained using the ONE Simulator [24]. Mobility traces used for the simulation were generated by the ONE Simulator and OpenJump [25] over a map depicting the area where the GOLDFISH Colombian tests took place, as can be seen in Figure 7 and Figure 8. The implementation of our algorithm in the ONE Simulator and the files used to run the simulations to compare it with other protocols in this scenario are available at the GitHub repository of this project [26]. Figure 9, Figure 10, Figure 11, Figure 12, Figure 13, Figure 14, Figure 15, Figure 16, Figure 17, Figure 18, Figure 19, Figure 20, Figure 21, Figure 22, Figure 23, Figure 24, Figure 25, Figure 26, Figure 27, Figure 28, Figure 29, Figure 30, Figure 31, Figure 32, Figure 33, Figure 34, Figure 35, Figure 36, Figure 37, Figure 38, Figure 39 and Figure 40 show simulation results.

**Figure 7:**
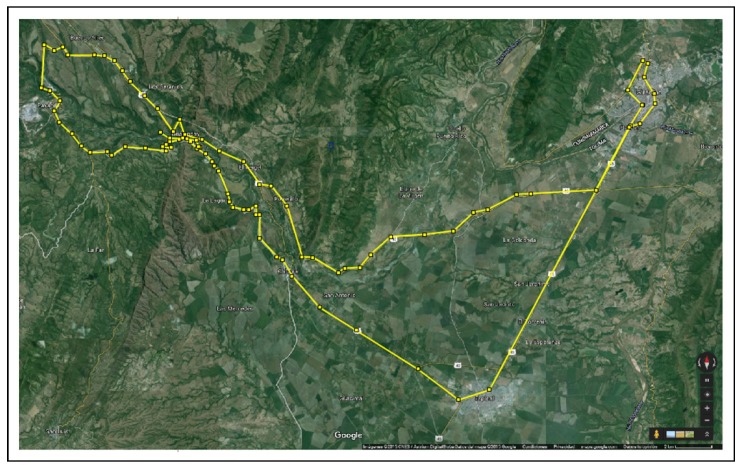
GOLDFISH project map from Google Earth in OpenJump.

**Figure 8:**
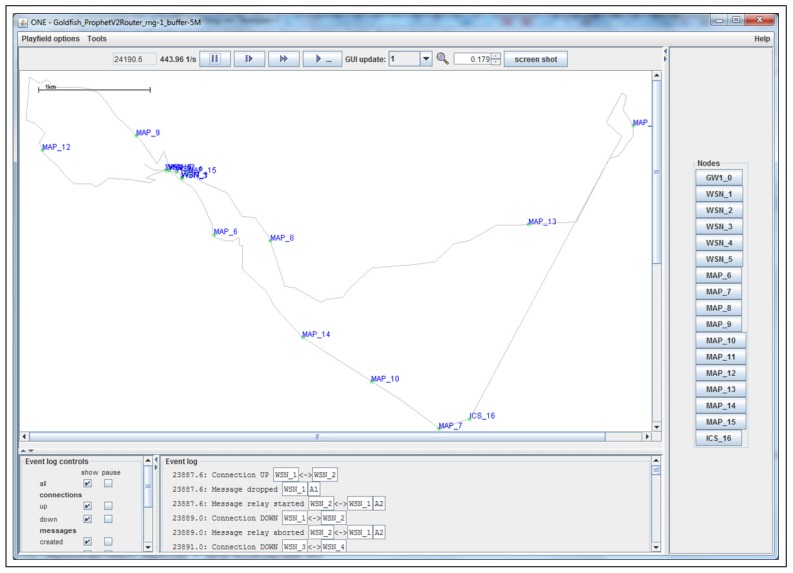
GOLDFISH project map scaled in the ONE Simulator.

**Figure 9:**
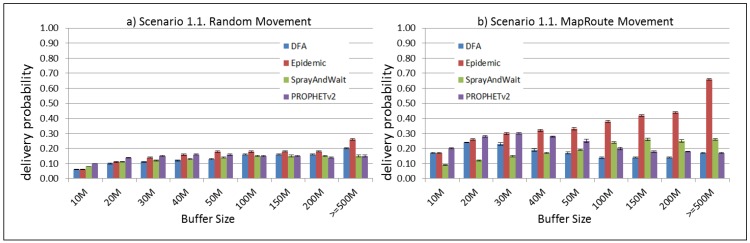
Results for scenario 1.1. Delivery probabilities with different Buffer Sizes, generating 2 MB messages every 1 to 2 min, (**a**) MAPs moving randomly on the path. (**b**) MAPs following the path in only one direction. DFA: Distributed Forwarding Algorithm.

**Figure 10:**
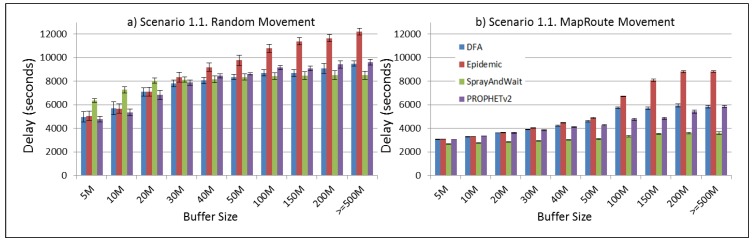
Results for scenario 1.1. Average delay in seconds with different Buffer Sizes, generating 2 MB messages every 1 to 2 min, (**a**) MAPs moving randomly on the path; (**b**) MAPs following the path in only one direction. DFA: Distributed Forwarding Algorithm.

**Figure 11:**
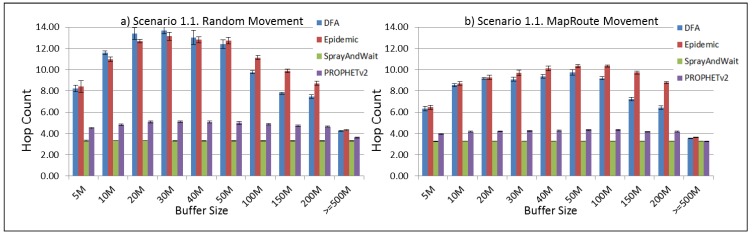
Results for scenario 1.1. Average hop count with different Buffer Sizes, generating 2 MB messages every 1 to 2 min, (**a**) MAPs moving randomly on the path; (**b**) MAPs following the path in only one direction. DFA: Distributed Forwarding Algorithm.

**Figure 12:**
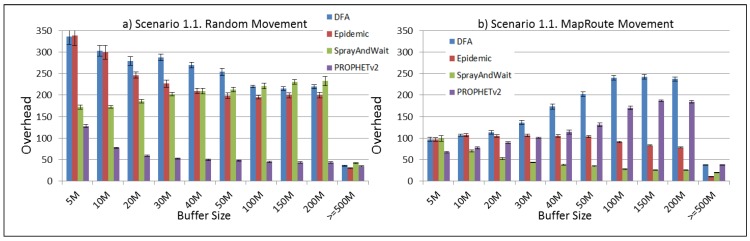
Results for scenario 1.1. Average overhead with different Buffer Sizes, generating 2 MB messages every 1 to 2 min, (**a**) MAPs moving randomly on the path; (**b**) MAPs following the path in only one direction. DFA: Distributed Forwarding Algorithm.

**Figure 13:**
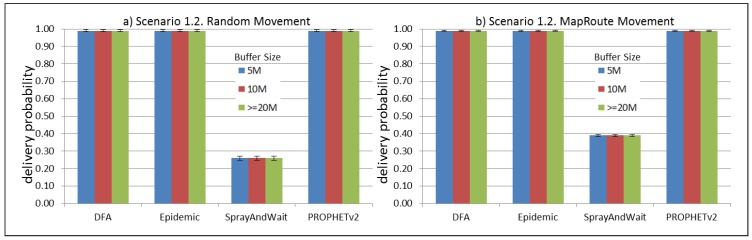
Results for scenario 1.2. Delivery probabilities with different Buffer Sizes, generating 2 kB messages every 1 to 2 min, (**a**) MAPs moving randomly on the path; (**b**) MAPs following the path in only one direction. DFA: Distributed Forwarding Algorithm.

**Figure 14:**
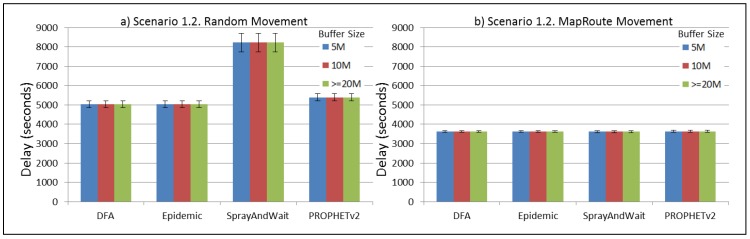
Results for scenario 1.2. Average delay in seconds with different Buffer Sizes, generating 2 kB messages every 1 to 2 min, (**a**) MAPs moving randomly on the path; (**b**) MAPs following the path in only one direction. DFA: Distributed Forwarding Algorithm.

**Figure 15:**
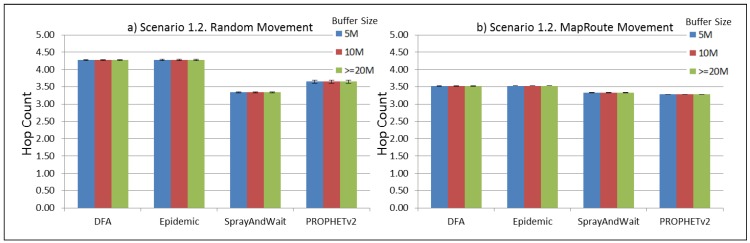
Results for scenario 1.2. Average hop count with different Buffer Sizes, generating 2 kB messages every 1 to 2 min, (**a**) MAPs moving randomly on the path; (**b**) MAPs following the path in only one direction. DFA: Distributed Forwarding Algorithm.

**Figure 16:**
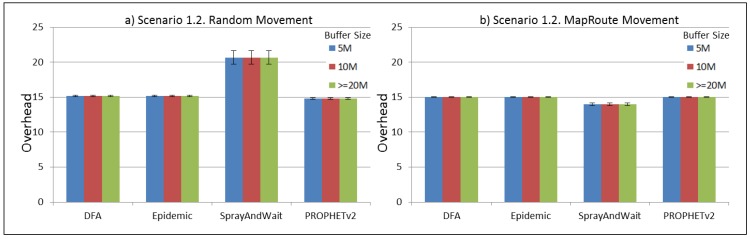
Results for scenario 1.2. Average overhead with different Buffer Sizes, generating 2 kB messages every 1 to 2 min, (**a**) MAPs moving randomly on the path; (**b**) MAPs following the path in only one direction. DFA: Distributed Forwarding Algorithm.

**Figure 17:**
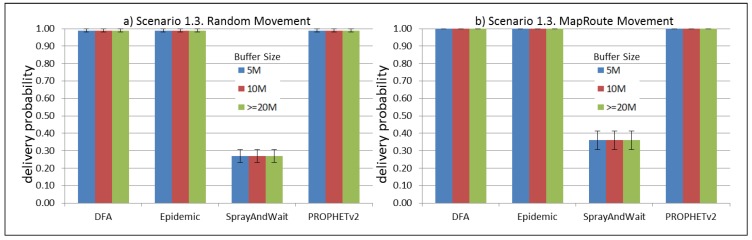
Results for scenario 1.3. Delivery probabilities with different Buffer Sizes, generating 2 kB messages every 1 to 2 h, (**a**) MAPs moving randomly on the path; (**b**) MAPs following the path in only one direction. DFA: Distributed Forwarding Algorithm.

**Figure 18:**
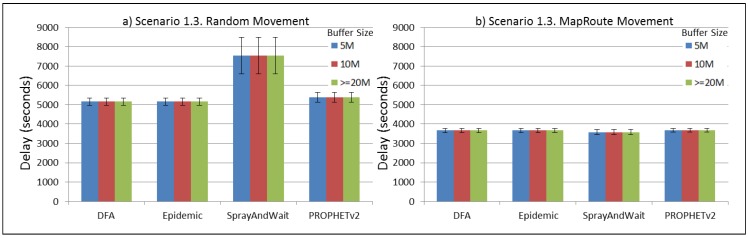
Results for scenario 1.3. Average delay in seconds with different Buffer Sizes, generating 2 kB messages every 1 to 2 h, (**a**) MAPs moving randomly on the path; (**b**) MAPs following the path in only one direction. DFA: Distributed Forwarding Algorithm.

**Figure 19:**
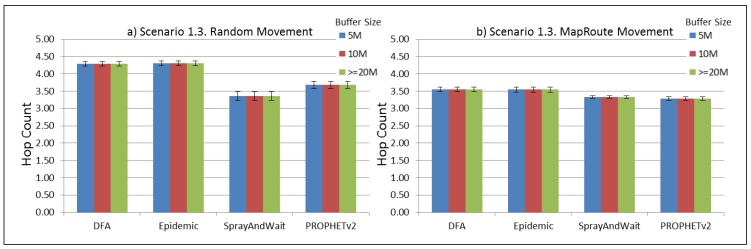
Results for scenario 1.3. Average hop count with different Buffer Sizes, generating 2 kB messages every 1 to 2 h, (**a**) MAPs moving randomly on the path; (**b**) MAPs following the path in only one direction. DFA: Distributed Forwarding Algorithm.

**Figure 20:**
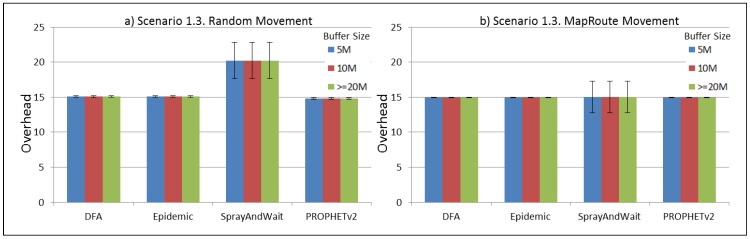
Results for scenario 1.3. Average overhead with different Buffer Sizes, generating 2 kB messages every 1 to 2 h, (**a**) MAPs moving randomly on the path; (**b**) MAPs following the path in only one direction. DFA: Distributed Forwarding Algorithm.

**Figure 21:**
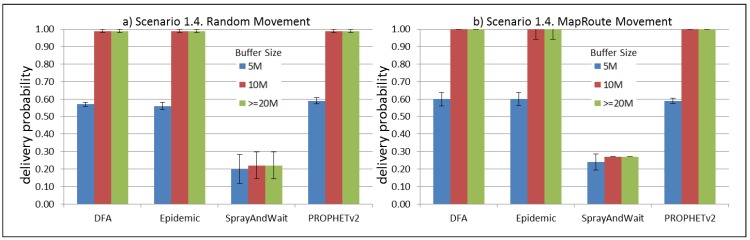
Results for scenario 1.4. Delivery probabilities with different Buffer Sizes, generating 2 MB messages every 1 to 2 h, (**a**) MAPs moving randomly on the path; (**b**) MAPs following the path in only one direction. DFA: Distributed Forwarding Algorithm.

**Figure 22:**
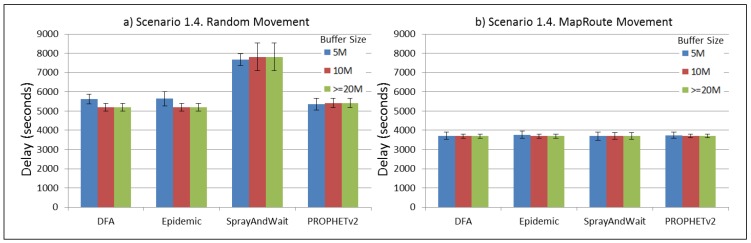
Results for scenario 1.4. Average delay in seconds with different Buffer Sizes, generating 2 MB messages every 1 to 2 h, (**a**) MAPs moving randomly on the path; (**b**) MAPs following the path in only one direction. DFA: Distributed Forwarding Algorithm.

**Figure 23:**
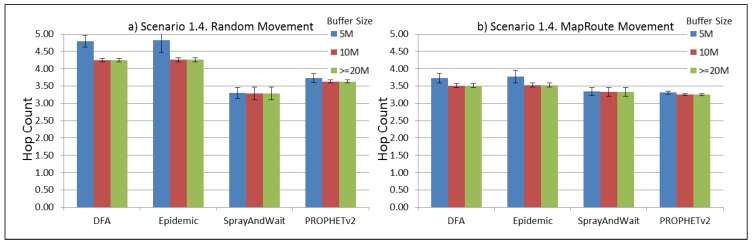
Results for scenario 1.4. Average hop count with different Buffer Sizes, generating 2 MB messages every 1 to 2 h, (**a**) MAPs moving randomly on the path; (**b**) MAPs following the path in only one direction. DFA: Distributed Forwarding Algorithm.

**Figure 24:**
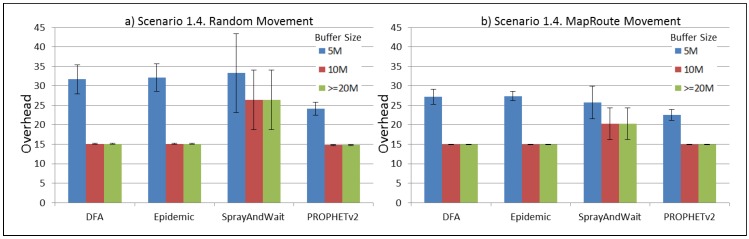
Results for scenario 1.4. Average overhead with different Buffer Sizes, generating 2 MB messages every 1 to 2 h, (**a**) MAPs moving randomly on the path; (**b**) MAPs following the path in only one direction. DFA: Distributed Forwarding Algorithm.

**Figure 25:**
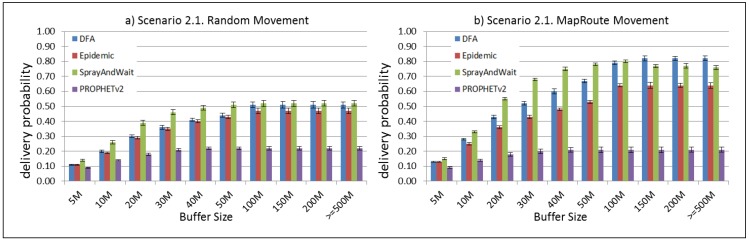
Results for scenario 2.1. Delivery probabilities with different Buffer Sizes, generating 2 MB messages every 1 to 2 min, (**a**) MAPs moving randomly on the path; (**b**) MAPs following the path in only one direction. DFA: Distributed Forwarding Algorithm.

**Figure 26:**
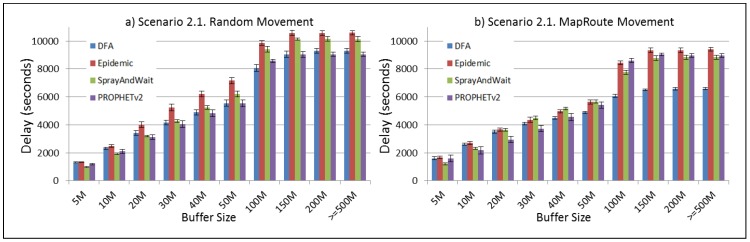
Results for scenario 2.1. Average delay in seconds with different Buffer Sizes, generating 2 MB messages every 1 to 2 min, (**a**) MAPs moving randomly on the path; (**b**) MAPs following the path in only one direction. DFA: Distributed Forwarding Algorithm.

**Figure 27:**
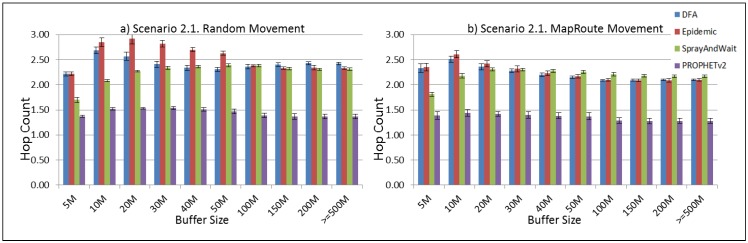
Results for scenario 2.1. Average hop count with different Buffer Sizes, generating 2 MB messages every 1 to 2 min, (**a**) MAPs moving randomly on the path; (**b**) MAPs following the path in only one direction. DFA: Distributed Forwarding Algorithm.

**Figure 28:**
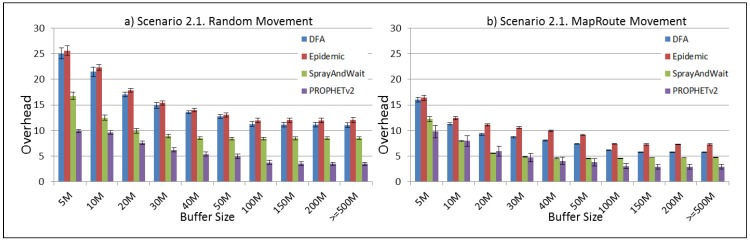
Results for scenario 2.1. Average overhead with different Buffer Sizes, generating 2 MB messages every 1 to 2 min, (**a**) MAPs moving randomly on the path; (**b**) MAPs following the path in only one direction. DFA: Distributed Forwarding Algorithm.

**Figure 29:**
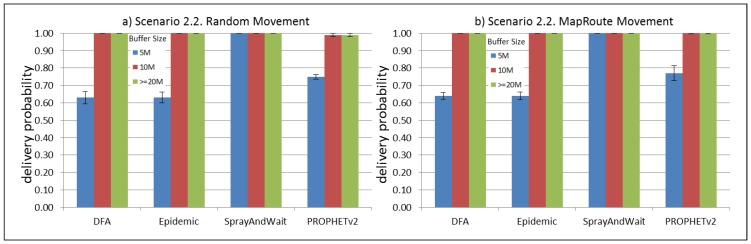
Results for scenario 2.2. Delivery probabilities with different Buffer Sizes, generating 2 kB messages every 1 to 2 min, (**a**) MAPs moving randomly on the path; (**b**) MAPs following the path in only one direction. DFA: Distributed Forwarding Algorithm.

**Figure 30:**
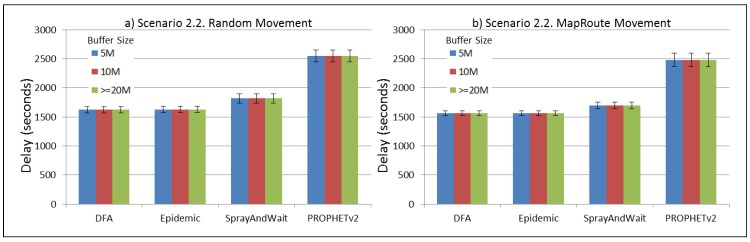
Results for scenario 2.2. Average delay in seconds with different Buffer Sizes, generating 2 kB messages every 1 to 2 min, (**a**) MAPs moving randomly on the path; (**b**) MAPs following the path in only one direction. DFA: Distributed Forwarding Algorithm.

**Figure 31:**
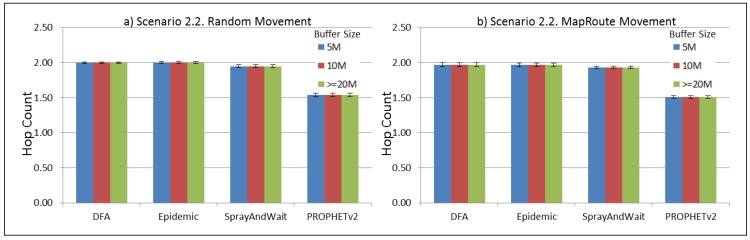
Results for scenario 2.2. Average hop count with different Buffer Sizes, generating 2 kB messages every 1 to 2 min, (**a**) MAPs moving randomly on the path; (**b**) MAPs following the path in only one direction. DFA: Distributed Forwarding Algorithm.

**Figure 32:**
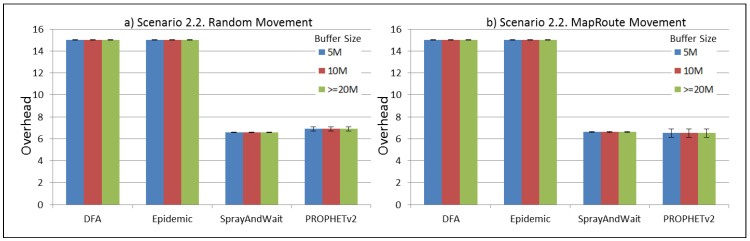
Results for scenario 2.2. Average overhead with different Buffer Sizes, generating 2 kB messages every 1 to 2 min, (**a**) MAPs moving randomly on the path; (**b**) MAPs following the path in only one direction. DFA: Distributed Forwarding Algorithm.

**Figure 33:**
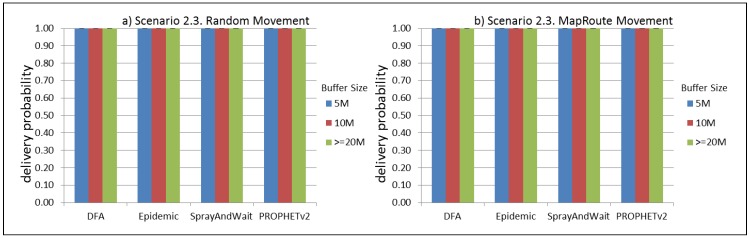
Results for scenario 2.3. Delivery probabilities with different Buffer Sizes, generating 2 kB messages every 1 to 2 h, (**a**) MAPs moving randomly on the path; (**b**) MAPs following the path in only one direction. DFA: Distributed Forwarding Algorithm.

**Figure 34:**
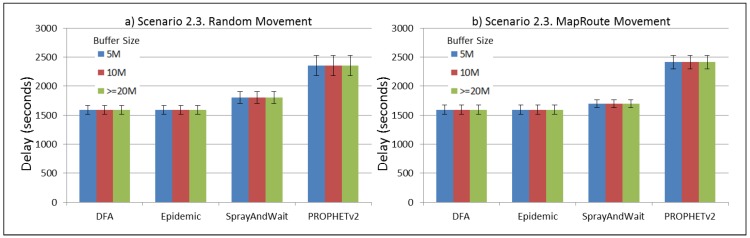
Results for scenario 2.3. Average delay in seconds with different Buffer Sizes, generating 2 kB messages every 1 to 2 h, (**a**) MAPs moving randomly on the path; (**b**) MAPs following the path in only one direction. DFA: Distributed Forwarding Algorithm.

**Figure 35:**
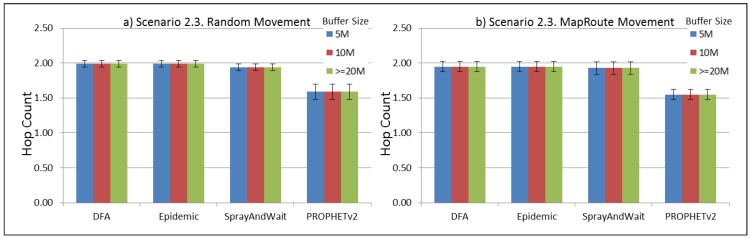
Results for scenario 2.3. Average hop count with different Buffer Sizes, generating 2 kB messages every 1 to 2 h, (**a**) MAPs moving randomly on the path; (**b**) MAPs following the path in only one direction. DFA: Distributed Forwarding Algorithm.

**Figure 36:**
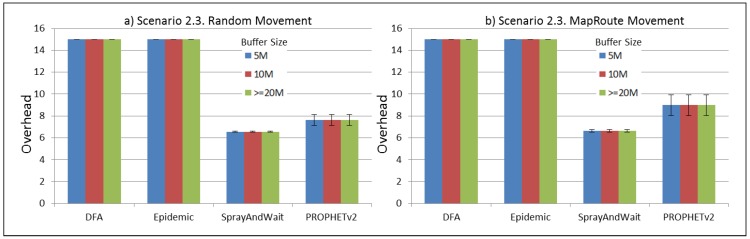
Results for scenario 2.3. Average overhead with different Buffer Sizes, generating 2 kB messages every 1 to 2 h, (**a**) MAPs moving randomly on the path; (**b**) MAPs following the path in only one direction. DFA: Distributed Forwarding Algorithm.

**Figure 37:**
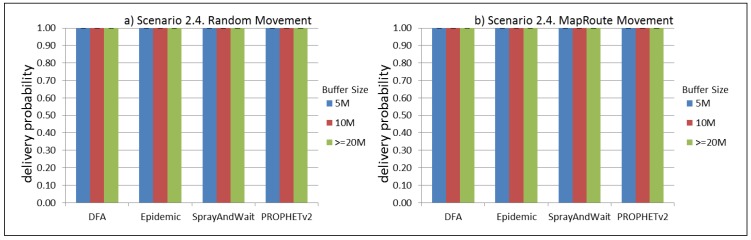
Results for scenario 2.4. Delivery probabilities with different Buffer Sizes, generating 2 MB messages every 1 to 2 h, (**a**) MAPs moving randomly on the path; (**b**) MAPs following the path in only one direction. DFA: Distributed Forwarding Algorithm.

**Figure 38:**
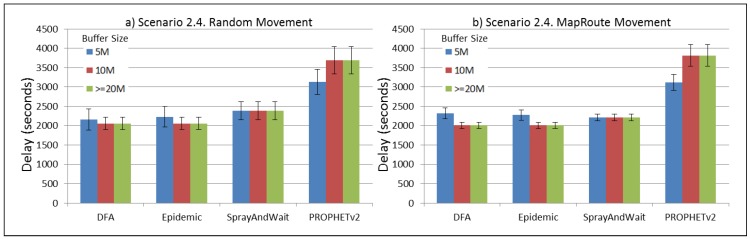
Results for scenario 2.4. Average delay in seconds with different Buffer Sizes, generating 2 MB messages every 1 to 2 h, (**a**) MAPs moving randomly on the path; (**b**) MAPs following the path in only one direction. DFA: Distributed Forwarding Algorithm.

**Figure 39:**
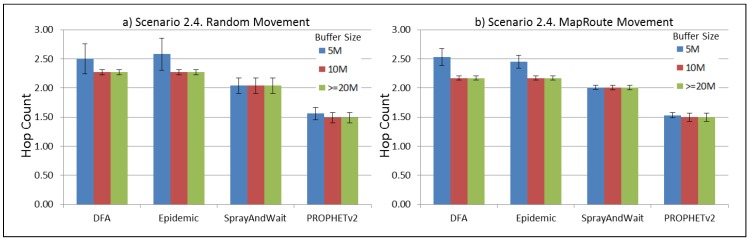
Results for scenario 2.4. Average hop count with different Buffer Sizes, generating 2 MB messages every 1 to 2 h, (**a**) MAPs moving randomly on the path; (**b**) MAPs following the path in only one direction. DFA: Distributed Forwarding Algorithm.

**Figure 40:**
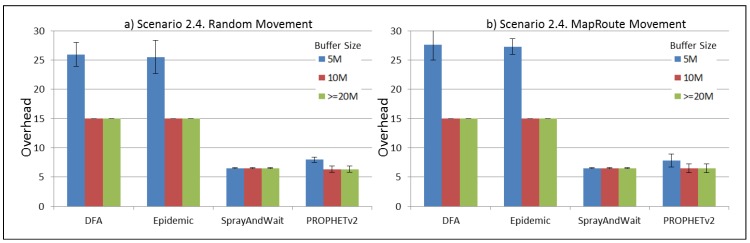
Results for scenario 2.4. Average overhead with different Buffer Sizes, generating 2 MB messages every 1 to 2 h, (**a**) MAPs moving randomly on the path; (**b**) MAPs following the path in only one direction. DFA: Distributed Forwarding Algorithm.

Our Distributed Forwarding Algorithm was tested against three of the most popular and used routing algorithms in DTNs, namely, Epidemic Routing [11], Spray and Wait [12], and PROPHETv2 [9]. Simulation parameters are showed in Table 3. We tested the scenario shown in Figure 7 and Figure 8 with different configurations, as shown in Table 4. In our first scenario, five sensor nodes (in the river) generate information messages that should be sent to the gateway (at the river bank), so it can pass them to the MAPs, and they can eventually reach the ICS. The number of nodes come from the GOLDFISH project, where there are a few nodes on the river communicating with the Gateway at the river shore, and the number of MAPs (10 mobile APs) was chosen based on the idea that there were enough transmission opportunities to take the sensor messages to the ICS and to cover a rural area of approximately 20 km × 16 km where public transportation is scarce. In this scenario, all sensor nodes’ messages should go through the Gateway; there is no direct communication from sensor nodes to MAPs. All nodes have wireless communication capabilities resembling IEEE 802.11b with 8 Mbps and a transmission range of 10 m. We also varied the movement model of the MAPs, from random around the path to MapRoute, which means that all MAPs followed the path at different speeds in only one direction. We tested the scenario with the four protocols, with 11 different buffer sizes (from 5 MB to 1 GB), with the two movement models for the MAPs, and with five different random seeds. Each scenario ran 440 times (once per every protocol, every buffer size, every movement model, and every random seed) for one simulated week (60,4800 s) to create the results (Figure 9, Figure 10, Figure 11, Figure 12, Figure 13, Figure 14, Figure 15, Figure 16, Figure 17, Figure 18, Figure 19, Figure 20, Figure 21, Figure 22, Figure 23 and Figure 24). Results are plotted for delivery rate, average delay, average hop count, and average overhead. After this, we ran another scenario setup (Scenario 2) where sensor nodes (message sources) could move randomly around all paths, creating contacts with MAPs and the ICS. In this second setup there is no need for a Gateway, since sensor nodes can communicate directly with the MAPs and the ICS. We also ran the same parameter combinations for this scenario (Figure 25, Figure 26, Figure 27, Figure 28, Figure 29, Figure 30, Figure 31, Figure 32, Figure 33, Figure 34, Figure 35, Figure 36, Figure 37, Figure 38, Figure 39 and Figure 40). The 95% confidence intervals were calculated for every result and they are plotted on the figures.

Results shown in Figure 9 indicate that, in this particular scenario, with low connectivity and a bottleneck node (the Gateway), DTN-based protocols can experience difficulties to deliver messages when information sources generate large messages (2 MB) at high frequency (less than 2 min between each message). The Gateway, then, becomes a mandatory path for all messages generating at the sensor nodes and seeking to reach the ICS through the MAPs. In the first scenario (Scenario 1.1, Figure 9), sensor nodes are sending 2 MB messages every 1 to 2 min, saturating the buffers and forcing nodes (probably the Gateway) to discard most messages, even with buffers over 500 MB per node. Results in Figure 13 show that when nodes generate light-sized messages (2 kB), DTN nodes can store more messages than in the previous scenario and deliver most of them. Figure 17 and Figure 21 show results for sensor nodes generating differently-sized messages every 1 to 2 h. Here, it can be seen that with this low frequency message generation, DTN-nodes can deliver most messages even with small-sized buffers. The exception in these results are for Spray and Wait protocol, but it can be due to configuration parameters and the bottleneck at the Gateway. The number of copies in Spray and Wait was chosen from simulation results from TheONE simulator, we used the file snw_comparison_settings.txt given in the simulator to compare different values of this parameter. From these simulations we deduced that for copy values over 8, in small sized scenarios, the delivery rate does not change considerably (less than 3 percent). We added this Spray and Wait comparison file to our GitHub project repository [26]. Figure 17 and Figure 21 show that our proposal performs at the same level or even better than Epidemic and PROPHETv2 protocols when the messages are generated every 1 to 2 h and the buffer size per node is equal to or greater than 10 MB. All results show that the delivery probability is slightly better when the MAPs follow the same direction at a constant speed than when they move randomly over the path.

Results for Scenario 2 (Sensor nodes moving randomly over all paths) show that our proposal performs as well as the other routing protocols or even better (in the case where large messages (2 MB) are generated every 1 to 2 min and the nodes have buffers with 100 MB or more). For the other sub-scenarios in Scenario 2, results indicate that all protocols deliver all or almost all messages when sensor nodes can freely move around the map and messages are small or are generated less frequently (1 to 2 h between each new message).

We can also see from Figure 10 that the delay acts as a non-decreasing function of the buffer size for all protocols in the first subscenario of Scenario 1. Spray and Wait shows the minor delay as the buffer increases, followed by PROPHETv2 and our DFA. For all other subscenarios of Scenario 1, all protocols show a very stable behaviour of their delay (Figure 14, Figure 18, and Figure 22). They experience less delay when the MAPs follow one direction in the map.

For Scenario 2, DFA has the best delay performance overall. It can be seen that even in the most demanding subscenario (Subscenario 2.1), DFA increases its delay as the buffer size increases but it does it at a lower rate than the other protocols. For all other subscenarios, the delay stays stable, with DFA and Epidemic having the minor delays, followed by Spray and Wait and PROPHETv2 with the bigger delays.

Regarding HopCount, we can see that for the first subscenario, DFA and Epidemic try to deliver more messages through more hops, PROPHET and Spray and Wait don’t change the number of hops when the buffer size changes (Figure 11). For the other subscenarios (Figure 15, Figure 19 and Figure 23), the hop count remains steady, with all protocols very close, but Spray and Wait having the least number of hops, followed by PROPHETv2 and then by DFA and Epidemic.

The hop count for all protocols in all subscenarios of Scenario 2 (Figure 27, Figure 31, Figure 35 and Figure 39) remains very stable. With PROPHETv2 using the minor number of hops and the other protocols having very similar figures between them.

Finally, for Scenario 1, overhead performance is plotted in Figure 12, Figure 16, Figure 20 and Figure 24. In the first subscenario (Figure 12), we can see that our DFA has the worst performance by replicating more messages than it is delivering. PROPHETv2 has the best performance when the MAPs move randomly, and Spray and Wait has the best performance when the MAPs move in only one direction. For all other subscenarios, all protocols have a very similar overhead performance, with Spray and Wait having a bigger overhead in general.

Regarding the overhead performance for Scenario 2, it can be seen from Figure 28, Figure 32, Figure 36 and Figure 40 that Spray and Wait and PROPHETv2 have the best performance for this metric, with similar figures, while DFA and Epidemic have a bigger overhead in general, with almost the same figures between them.

## 5. Conclusions

Delay/Disruption Tolerant Networks are a promising technology for rural internet connectivity applications. They can connect people in remote places using non-real-time applications at low costs. Since DTNs can use local transportation infrastructure, they can reach most regions where no networking or even communications infrastructure exists. For our application, we have shown that DTNs are a feasible way to retrieve sensor data from an isolated spot if we can have a vehicle or a set of vehicles that eventually or periodically will reach that spot.

Simulation results show that our Distributed Forwarding Algorithm can deliver most messages (around 99%) using delivery probabilities learned from neighboring nodes, contact history information and replication, and a buffer of 10 MB or more. In the most demanding sub-scenarios, those where 2 MB messages were generated every 1 to 2 min, all protocols performed similarly. However, when we increased the connectivity in the network, giving sensor nodes the opportunity to directly contact the MAPs or the ICS, our proposed algorithm performed better on delivery rate and delay than all other protocols for buffer values over 100 MB.

Due to our proposed DFA implementation of replication and its slow updating of transient delivery probabilities, it uses more hops trying to get the message to its destination and it generates more overhead than Spray and Wait and PROPHETv2, in most cases.

Our algorithm can be used in applications like GOLDFISH where data is taken from a remote site and decision making takes place in another location. We are looking at applications where those decisions do not have to be taken instantly with a single event, but over a lapse of time with historical data—applications like pollution monitoring.

Future work includes researching tuning parameters for the algorithm and a better way to use the contact information to schedule transmission or look for alternative routes. Also, we look forward to performing real tests with the necessary hardware and mobile nodes in a real scenario.

## Figures and Tables

**Table 1:** Related protocols.

Characteristics	Protocols
Flooding-based	Epidemic [11], Spray and Wait [12]
Social-based	SimBet [15], BUBBLE [13], and PeopleRank [16]
Hybrid protocols	PROPHET [9], MPAD [14] RAPID [17], LSF [19] and SMART [18]

**Table 2:** Mathematical model parameters.

Parameter	Definition
*N*	Set of nodes
*E*	Set of edges
*P*	Set of candidate paths
*T*	Set of discrete time values
$c_{ij}$	Capacity of edge (i,j)∈E
$c_{ii}$	Storage capacity of node *i*
$a_{ij}\left(t\right)$	Availability Probability of edge (i,j)∈E at time *t*. $a_{ij}\left(t\right)=a_{ji}\left(t\right)$
$d_{ij}\left(t\right)$	Delivery Probability of node *i* through node *j* at time *t*
$b_{i}\left(t\right)$	Set of offers or demands from node *i* at time *t*
*t*	discrete time value, where t∈T
$\delta_{t}$	Time interval duration
*v*	number of time values since last update
$x_{ij}\left(t\right)$	Decision variable. How much data flows through edge (i,j)∈E at time *t*

**Table 3:** Simulation parameters. ICS: Internet Connected Server. MAP: Mobile Access Point.

Parameter	Definition
Gateway	1 node
Sensor Nodes	5 nodes
ICS	1 node (destination node)
Mobile APs	10 nodes going through the defined path
RandomMvmt	MAPs going through the path in a random fashion
MapRoute	MAPs going through the path following one direction
Buffer Size	Going from 5 MB to 1000 MB. How much information a node can hold
Message Size	Size of each message. It goes from 2 kB to 2 MB
Message Interval	It determines how often messages are generated
SnWCopies	Spray and Wait copies set to 8
DFA	Distributed Forwarding Algorithm (Our proposal)

**Table 4:** Simulation scenarios. X = 1. Wireless Sensor Network (WSN) nodes in the river. X = 2. WSN nodes all around the map.

Parameter	Subscenario X.1	Subscenario X.2	Subscenario X.3	Subscenario X.4
Message Size	2 MB	2 kB	2 kB	2 MB
Message Interval	1–2 min	1–2 min	1–2 h	1–2 h

## References

[1] Rzeszutko E., Solano-Donado F., Mycek M. FP7 Project Goldfish—Practical Application of Sensor Networks for Watercourse Pollution Monitoring. Proceedings of the XXX Krajowe Sympozjum Telekomunikacji i Teleinformatyki (KSTiT’2014).

[2] Cerf V., Burleigh S., Hooke A., Torgerson L., Durst R., Scott K., Fall K., Weiss H. (2007). Delay-tolerant networking architecture. IETF Trust.

[3] Berners-Lee T., Fielding R., Masinter L. (2005). Uniform Resource Identifier (URI): Generic Syntax. Internet Soc..

[4] Scott K. L., Burleigh S. Bundle Protocol Specification. https://tools.ietf.org/html/rfc5050.

[5] Project Loon Google Inc. https://www.google.com/loon/.

[6] Internet.org Facebook Inc. https://www.internet.org/projects.

[7] Pentland A., Fletcher R., Hasson A. (2004). Daknet: Rethinking connectivity in developing nations. Computer.

[8] Watkins J., Tacchi J., Kiran M.S. The Role of Intermediaries in the Development of Asynchronous Rural Access. Universal Access in Human-Computer Interaction. Applications and Services, Proceedings of the 5th International Conference, UAHCI 2009, Held as Part of HCI International 2009.

[9] Lindgren A., Doria A., Davies E., Grasic S. Probabilistic Routing Protocol for Intermittently Connected Networks. https://tools.ietf.org/html/rfc6693.

[10] Gomez C., Paradells J. (2015). Urban Automation Networks: Current and Emerging Solutions for Sensed Data Collection and Actuation in Smart Cities. Sensors.

[11] Vahdat A., Becker D. (2000). Epidemic Routing for Partially Connected Ad Hoc Networks.

[12] Spyropoulos T., Psounis K., Raghavendra C.S. Spray and Wait: An Efficient Routing Scheme for Intermittently Connected Mobile Networks. Proceedings of the 2005 ACM SIGCOMM workshop on Delay-tolerant networking.

[13] Hui P., Crowcroft J., Yoneki E. BUBBLE Rap: Social-based Forwarding in Delay Tolerant Networks. Proceedings of the ACM MobiHoc’08.

[14] Zhu J., Cao J., Liu M., Zheng Y., Gong H., Chen G. A Mobility Prediction-Based Adaptive Data Gathering Protocol for Delay Tolerant Mobile Sensor Network. Proceedings of the Global Telecommunications Conference, 2008, (IEEE GLOBECOM 2008).

[15] Daly E.M., Haahr M. Social network analysis for routing in disconnected delay-tolerant manets. Proceedings of the ACM MobiHoc’07.

[16] Mtibaa A., May M., Diot C., Ammar M. Peoplerank: Social opportunistic forwarding. Proceedings of the IEEE INFOCOM’10.

[17] Balasubramanian A., Levine B., Venkataramani A. (2010). Replication routing in DTNs: A resource allocation approach. IEEE ACM Trans. Netw..

[18] Tang L., Zheng Q., Liu J., Hong X. Smart: A selective controlled flooding routing for delay tolerant networks. Proceedings of the Fourth International Conference on Broadband Communications, Networks and Systems, 2007, BROADNETS 2007.

[19] Spyropoulos T., Turletti T., Obraczka K. (2009). Routing in delay-tolerant networks comprising heterogeneous node populations. IEEE Trans. Mob. Comput..

[20] Cao Y., Sun Z. (2013). Routing in Delay/Disruption Tolerant Networks: A Taxonomy, Survey and Challenges. IEEE Commun. Surv. Tutor..

[21] Khabbaz M.J., Assi C.M., Fawaz W.F. (2012). Disruption-Tolerant Networking: A Comprehensive Survey on Recent Developments and Persisting Challenges. IEEE Commun. Surv. Tutor..

[22] Psaras I., Wood L., Tafazolli R. (2010). Delay-Disruption-Tolerant Networking: State of the Art and Future Challenges.

[23] Velásquez-Villada C., Solano F., Donoso Y. (2015). Routing Optimization for Delay Tolerant Networks in Rural Applications Using a Distributed Algorithm. Int. J. Comput. Commun. Control.

[24] Keränen A., Ott J., Kärkkäinen T. The ONE Simulator for DTN Protocol Evaluation. Proceedings of the 2nd International Conference on Simulation Tools and Techniques (ICST).

[25] OpenJump GIS (2016). http://www.openjump.org/.

[26] Velásquez-Villada C. DFA Project Repository (2016). https://github.com/cevelasquezv/.

